# Toll-Like Receptors Agonists: First-Line Defense Tools in the Pandemic Preparedness Arsenal?

**DOI:** 10.34172/aim.31792

**Published:** 2024-11-01

**Authors:** Farrokh Habibzadeh, Parham Habibzadeh, Mihai G. Netea, Mahboobeh Yadollahie

**Affiliations:** ^1^Independent Research Consultant, Shiraz, Iran; ^2^Department of Medicine, University of Pittsburgh Medical Center, Pittsburgh, Pennsylvania, USA; ^3^Radboud Center for Infectious Diseases, Department of Internal Medicine, Radboud University Medical Center, Nijmegen, Netherlands; ^4^Department of Immunology and Metabolism, Life and Medical Science Institute, University of Bonn, Bonn, Germany; ^5^Independent Researcher, Shiraz, Iran

## Introduction

 In 1921, two French scientists, Albert Calmette and Camille Guérin, developed the first vaccine against tuberculosis, BCG.^1^ The vaccine contains a live-attenuated bacterium. In 1931, Calmette reported that children vaccinated with BCG at birth were approximately 75% less likely to die of any cause in their early years of life.^2^ The observed protective effect conferred by the vaccine was disproportionally greater than that one might have estimated solely based on the incidence of tuberculosis in that period. Later on, in the 1960s and 1970s, similar observations were made by two virologists practicing in the former Soviet Union, Marina Voroshilova and Mikhail Chumakov; they found that individuals who had been vaccinated with oral polio vaccine (OPV) were 70% less likely to develop acute influenza or other respiratory infections.^3^ In 1978, Peter Aaby studied the causes of high mortality rate among children in Guinea-Bissau. The next year, in 1979, when a measles outbreak hit a district in the capital city of Bissau, Aaby started vaccinating children and also observed that the vaccine reduced overall child mortality by around 50%; the decrease in mortality rate was much higher than would be anticipated if the vaccine was preventing deaths from measles alone. A systematic review including meta-analysis of 10 studies conducted in Bangladesh, Benin, Burundi, Guinea-Bissau, Haiti, Senegal, and Zaire (now, the Democratic Republic of the Congo) confirmed that the MMR (measles, mumps and rubella) vaccine can also confer non-specific protection against unrelated infections.^4^ An ecological study soon conducted after the COVID-19 pandemic, reveals that the cumulative incidence of COVID-19 in countries using OPV is significantly lower than countries where only the inactivated polio vaccine is used.^5^ In a cohort study on more than 4000 women, it was shown that mothers indirectly exposed to the live-attenuated polio virus existing in OPV are significantly more resistant to SARS-CoV-2 compared with a group of matched women without the exposure.^6^ A recent randomized clinical trial has also confirmed that OPV provides non-specific protection against SARS-CoV-2.^7^

 These epidemiological data argue that BCG, OPV, and MMR, all live-attenuated vaccines, not only provide long-term specific immunity against their target diseases, but also confer short-term non-specific protection against many unrelated infections. It has been hypothesized that this non-specific short-term protection is attributed to the stimulation of innate immune system responsiveness, so-called trained immunity.^8,9^ However, no solid evidence is yet available to support induction of trained immunity as a correlate of protection.

## Toll-Like Receptor-7, -8, and -9 and Protection Against SARS-CoV-2

**Figure 1:**
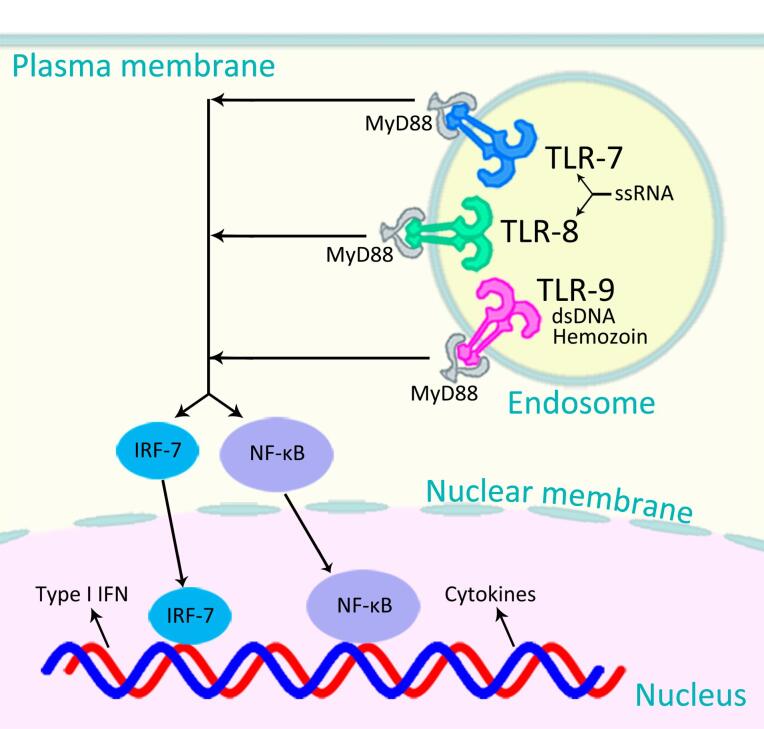
Signaling Pathways of TLR-7, -8, and -9. TLR-7 and -8 are triggered by viral ssRNA; TLR-9, by bacterial, viral, and plasmodium dsDNA and hemozoin. Triggering of each of these TLRs results in activation of either MyD88–IRF-7 pathway to produce type I IFN, or MyD88–NF-κB pathway to produce cytokines.^10^ TLR: Toll-like receptor; ssRNA: single-stranded RNA; dsDNA: double-stranded DNA; MyD88: myeloid differentiation primary response 88; IFN: interferon; IRF-7: IFN regulatory factor 7; and NF-κB: nuclear factor kappa-light-chain-enhancer of activated B cells

 Innate immunity is the first line of defense against foreign entities including bacteria, viruses, and parasites. It generally acts through production of non-specific pro-inflammatory responses.^8^ The mechanism involved in discrimination of self from non-self mostly relies on pattern recognition receptors, such as Toll-like receptors (TLRs). TLRs are integral membrane-bound proteins that are triggered by a variety of pathogen-associated molecular patterns, including microbial RNA and DNA fragments.^10,11^ For example, TLR-7 and -8 are triggered by viral single-stranded RNA; TLR-9, by bacterial, viral, and plasmodium double-stranded DNA and hemozoin (Figure 1). Triggering of TLR-7, -8, and -9 results in activation of myeloid differentiation primary response 88 (MyD88) and a cascade of certain intracellular downstream signaling reactions, mainly through interferon (IFN) regulatory factor 7 (IRF-7) and nuclear factor kappa-light-chain-enhancer of activated B cells (NF-κB) pathways, leading to production of type I IFNs such as IFN-α and -β, type II IFN (IFN-γ), and pro-inflammatory cytokines such as interleukin-6 (IL-6), IL-1β, and tumor necrosis factor-α (TNF-α) (Figure 1).^10,11^ We hypothesize that stimulation of TLR-7, -8, or -9 (by their agonists) would confer temporary non-specific protection against SARS-CoV-2 and, probably other infections, even emerging ones.

 The hypothesis that live-attenuated vaccines could provide short-term non-specific protection against unrelated infections is supported by the data that the live-attenuated polio virus existing in the OPV, containing single-stranded RNA with the ability to stimulate TLR-7 and -8, results in production of pro-inflammatory cytokines and type I IFNs and leads to temporary protection against influenza (as reported by Voroshilova and Chumakov^3^) and SARS-CoV-2 (as reported by Habibzadeh et al).^5,6^ The pathogenesis of polio virus, influenza virus, and SARS-CoV-2 infections are quite different. The only thing they share is that they are all RNA viruses with the ability to stimulate TLR-7 and -8. If the non-specific protection provided by OPV (the live-attenuated polio virus) against influenza and SARS-CoV-2 viruses is through stimulation of TLR-7 and -8, it might also confer protection against another RNA virus, *e.g.*, the human immunodeficiency virus (HIV).

 Mother-to-child transmission of HIV can occur during three phases: *in utero*, when maternal viral particles can cross the placenta to infect the fetus; during the labor and delivery, when the neonate is being exposed to the infected maternal blood and secretions in the birth canal; and after the birth, when the virus within the mother milk (mainly within susceptible cells such as infected lymphocytes) can infect the child.^12^ For the daily exposure to breast milk over a long period (several months after birth), the cumulative risk of the transmission during this period is high, such that breastfeeding accounts for around 40% of all mother-to-child transmission of HIV.^13^ If OPV could really confer protection against HIV, it has to decrease the cumulative incidence of mother-to-child transmission of HIV after the birth in countries where OPV is part of their national immunization. And, it seems that it really does; in an ecological study, we showed that after controlling for confounders, vaccination with OPV (containing live-attenuated polio virus) compared with inactivated polio vaccine (no live virus), is independently associated with lower rate of mother-to-child transmission of HIV.^14^

 The signaling pathway after the stimulation of TLR-7 and -8 (by single-stranded RNA viruses such as SARS-CoV-2, influenza, and HIV) is very similar to that of TLR-9 (stimulated by DNA and hemozoin) which ultimately results in production of pro-inflammatory cytokines and type I IFN (Figure 1).^10^ If the underlying mechanism is the stimulation of the innate immune system (mainly through the stimulation of TLR-7 and -8, for the viruses mentioned above), rather than the similarity in genetic materials of the pathogens (all RNA viruses), then stimulation of TLR-9, which although is triggered by DNA and hemozoin (not RNA), activates a very similar cascade of reactions to produce the same pro-inflammatory cytokines and type I IFN (Figure 1), should also confer degrees of protections against the above-mentioned RNA viruses.

 It has been shown that malaria can induce a robust innate immune response.^15^ The parasite crystal hemozoin coated with its genomic DNA activates TLR-9 (Figure 1).^16^ In a recent study on 53 African countries, it has been shown that wherever in Africa the prevalence of malaria is high, the cumulative incidence of COVID-19 is relatively low, and *vice versa*, reflecting that malaria (presumably, through the stimulation of TLR-9 by its DNA and hemozoin^16^) might also protect people against SARS-CoV-2,^17^ probably in a similar way that OPV does.

 If stimulation of TLR-7 and -8 by OPV, and TLR-9 by malaria confers temporary non-specific protection against an array of infections, then direct stimulation of these receptors by their specific synthetic agonists should also do so.

 Administration of live, even attenuated, pathogens may be associated with certain side-effects, sometimes serious. For example, although very rare, use of OPV may be associated with development of vaccine-associated paralytic polio. Another potential side-effect of OPV use is that the virus in the vaccine can mutate and become circulating vaccine-derived poliovirus that may cause small outbreaks of paralytic disease.^18,19^ Many developed countries have abandoned the use of OPV; they use inactivated polio vaccine instead. Therefore, it is preferable to use TLR-7, -8, or -9 mimetics, rather than live-attenuated pathogens in order to stimulate the innate immune system to provide the non-specific protection mentioned above.

## Discussion

 Over the past decades, we have witnessed several emerging infectious diseases including HIV, severe acute respiratory syndrome (SARS), Middle East respiratory syndrome (MERS), and lately, the SARS-CoV-2. When an emerging infectious disease occurs, it would normally take around 12–24 months to conduct the necessary pre-clinical studies and clinical trials, and manufacture an effective specific vaccine; it just takes 3–4 months to complete the required studies to test broadly-specific vaccines.^9^ Furthermore, if the emerging pathogen possesses a high rate of mutation (like SARS-CoV-2), the effectiveness of the specific vaccine developed might be highly diminished. Under such circumstances, having a means to produce non-specific protection, even temporary, would be of paramount importance and life-saving.

 Clinical application of many TLR agonists have been studied so far. For example, imiquimod, an FDA-approved TLR-7 agonist, is used to treat genital warts and non-melanoma skin cancers.^20^ Treatment with monoclonal antibodies has been proposed for the treatment of different types of cancers. TLR-7 and -8 agonists have been used for improving the antibody-dependent cellular cytotoxicity for the treatment of various malignancies.^21^ Several TLR-9 agonists have also been studied for the treatment of cancer and infectious diseases.^22,23^ A systematic review of 9 single-arm studies and 12 controlled trials revealed that the toxicity of TLR-9 agonists is generally low.^24^ TLR-7, -8, and -9 have also been used as adjuvants in vaccines.^25,26^ TLR-7, -8, and -9 agonists, through stimulation of innate immune system, may confer temporary non-specific protection against emerging infectious diseases. They can be used either as broadly-specific vaccines, instead of live-attenuated vaccines, or as adjuvants in vaccines. If the proposed hypothesis is correct, then we expect that those receiving these TLR agonists (*e.g.*, imiquimod or vidutolimod^27^), for instance, for treating their cancer, carry a lower risk of developing many infectious diseases, as an example, COVID-19, compared with a matched group of patients not receiving these TLR agonists. The route of administration of these agonists would be very important. It is necessary to test the safety profile of these agonists when administered locally in the mucosa of respiratory tract, which would be probably the preferred route of administration, or through other routes. This hypothesis should be tested in well-controlled studies. If the hypothesis is proved, TLR-7, -8, and -9 agonists may become the first-line defense tools in the pandemic preparedness arsenal.
